# Collective Cell Migration on Collagen-I Networks: The Impact of Matrix Viscoelasticity

**DOI:** 10.3389/fcell.2022.901026

**Published:** 2022-07-04

**Authors:** Ivana Pajic-Lijakovic, Milan Milivojevic, Andrew G. Clark

**Affiliations:** ^1^ University of Belgrade, Faculty of Technology and Metallurgy, Belgrade, Serbia; ^2^ University of Stuttgart, Institute of Cell Biology and Immunology, Stuttgart, Germany; ^3^ University of Stuttgart, Stuttgart Research Center Systems Biology, Stuttgart, Germany; ^4^ University of Tübingen, Center for Personalized Medicine, Tübingen, Germany

**Keywords:** extracellular matrix, viscoelasticity, cell rearrangement, matrix remodeling, residual stress accumulation, collective cell migration, collagen

## Abstract

Collective cell migration on extracellular matrix (ECM) networks is a key biological process involved in development, tissue homeostasis and diseases such as metastatic cancer. During invasion of epithelial cancers, cell clusters migrate through the surrounding stroma, which is comprised primarily of networks of collagen-I fibers. There is growing evidence that the rheological and topological properties of collagen networks can impact cell behavior and cell migration dynamics. During migration, cells exert mechanical forces on their substrate, resulting in an active remodeling of ECM networks that depends not only on the forces produced, but also on the molecular mechanisms that dictate network rheology. One aspect of collagen network rheology whose role is emerging as a crucial parameter in dictating cell behavior is network viscoelasticity. Dynamic reorganization of ECM networks can induce local changes in network organization and mechanics, which can further feed back on cell migration dynamics and cell-cell rearrangement. A number of studies, including many recent publications, have investigated the mechanisms underlying structural changes to collagen networks in response to mechanical force as well as the role of collagen rheology and topology in regulating cell behavior. In this mini-review, we explore the cause-consequence relationship between collagen network viscoelasticity and cell rearrangements at various spatiotemporal scales. We focus on structural alterations of collagen-I networks during collective cell migration and discuss the main rheological parameters, and in particular the role of viscoelasticity, which can contribute to local matrix stiffening during cell movement and can elicit changes in cell dynamics.

## Introduction

During cell migration and tissue rearrangement, cells exert mechanical forces on their substrate via the cytoskeleton, which is coupled to the substrate via adhesions. This mechanical force results in a local strain, or deformation, of the substrate. Collagen-I networks are a common biological substrate that can facilitate single-cell or collective modes of migration and that can be deformed by cell-generated stresses (Clark and Vignjevic, 2015). Collagen networks are formed from triple-helical strands of collagen polypeptides that self-assemble into larger fibers and crosslinked, overlapping network structures (Shoulders and Raines, 2009). Two features of collagen network rheology that are important for cell-ECM interactions are strain stiffening and residual stress accumulation in response to strain. Both of these features are related to the degree of network plasticity, which is governed by a number of factors including: filament conformations, alignment, density and cross-linking/connectivity and also depend on the magnitude of strain and strain rate during extension or compression (Ban et al., 2018; Jansen et al., 2018).

The topological and rheological properties of collagen networks and local structural changes in the network can, in turn, feed back onto cellular activity. The impact of several factors such as fiber length and diameter (Sapudom et al., 2015; Sarker et al., 2019), fiber alignment (Dickinson et al., 1994; Conklin et al., 2011; Riching et al., 2014; Fraley et al., 2015), network pore size (Wolf et al., 2013), network stiffness (Shi et al., 2014) and network viscoelasticity (Clark et al., 2020; Clark et al., 2022; Elosegui-Artola et al., 2022) have been shown experimentally to affect single-cell and collective migration dynamics on collagen networks. Moreover, changes in the viscoelastic properties of ECM networks have also been linked to pathologies, including brain, breast and liver cancer (Sinkus et al., 2005; Shahryari et al., 2019; Chaudhuri et al., 2020; Kaspar-Josche et al., 2020). In addition, changes in collagen cross-linking, most prominently by lysyl oxidase (LOX) has also been associated with increased cancer metastasis (Levental et al., 2009; Cox et al., 2013).

Numerous previous studies have developed theoretical models to predict collagen network behavior during mechanical stress (Broedersz and MacKintosh, 2014; Ban et al., 2018; Dietrich et al., 2018; Jansen et al., 2018). However, the precise effects of local network remodeling on cell migration and tissue rearrangements are still unclear. The main goal of this review is to describe the time-scales of collagen-I network structural changes under complex strain conditions generated by cell movement, to identify the key parameters responsible for the network stiffening and to discuss the effects of collagen network structure on cell dynamics.

### Cellular Reorganization of ECM Networks

Groups of cells generate shear and volumetric strain on collagen networks during collective migration (Gjorevski and Nelson, 2012). The manner in which the network is modified is influenced both by the mechanical stress generated by cells and the internal rheological response of the collagen networks themselves and must be considered on several scales (Sander et al., 2009). Cellular mechanical stresses on the substrate are primarily the result of actomyosin-based contraction and coupling with the underlying substrate by cell-ECM adhesions. These forces are caused by cumulative effects of various cellular processes at time-scales of minutes to hours, including cell polarization (Alert et al., 2019), cadherin turnover (Lee and Wolgemuth, 2011) and actomyosin-dependent traction forces (Thoumine and Ott, 1997). The mechanical stresses exerted by cells can lead to long-range deformations in ECM networks over several hours, which can influence cell behavior across distances orders of magnitude larger than the size of single cells (Shi et al., 2014; Hall et al., 2016; Pakshir et al., 2019). In addition, information flow during intra- and intercellular signaling (sensing, signal transduction, gene expression) can also occur on various timescales (Petrungaro et al., 2019). Together, these factors and can lead to accumulation of stresses during collective migration on time-scales of several hours (Pajic-Lijakovic and Milivojevic, 2019, 2020).

**FIGURE 1:**
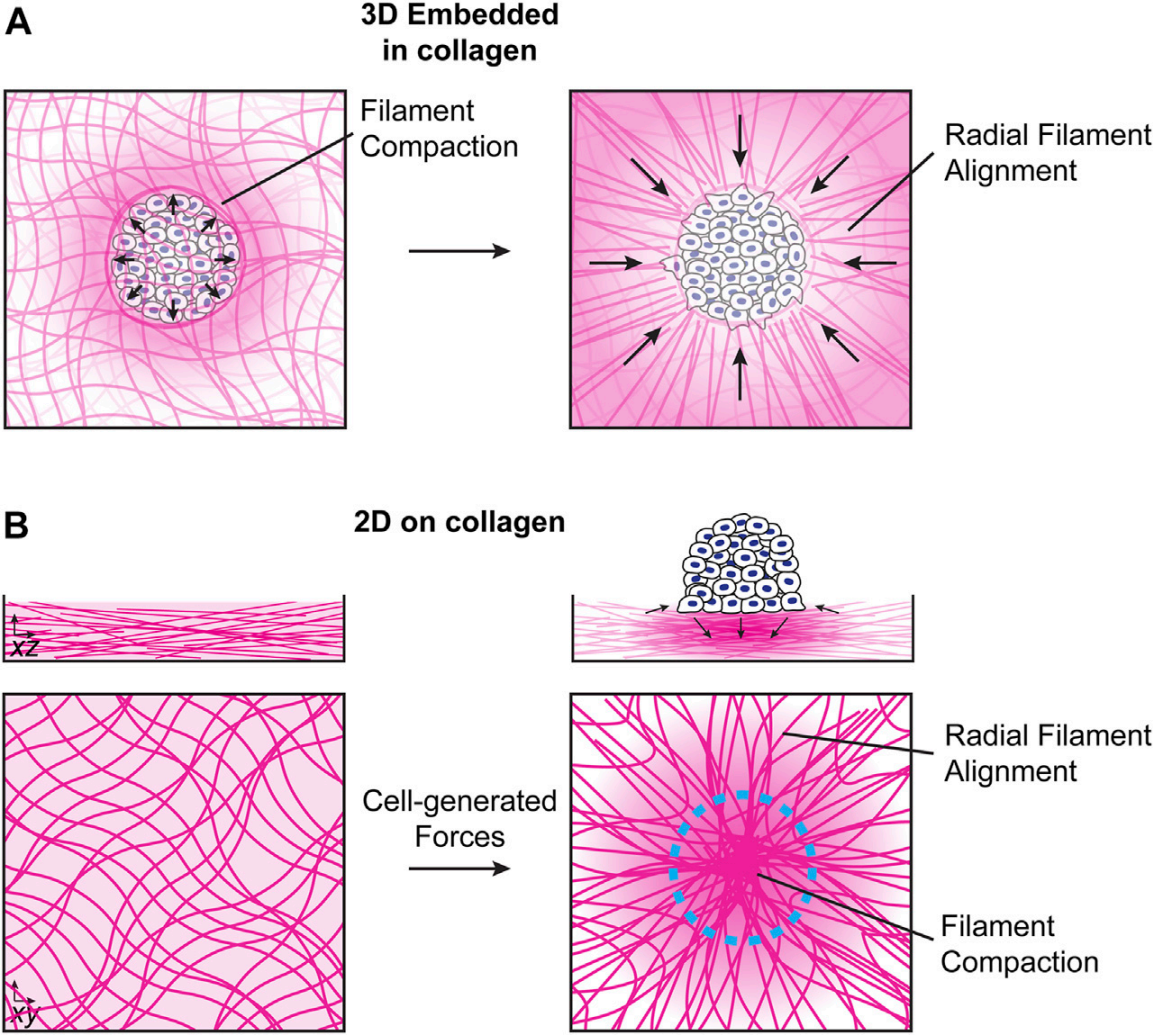
Local collagen network reorganization by cell clusters. **(A).** For clusters embedded in 3D collagen networks, cells initially push on the collagen network, leading to filament compaction and compression of the network at the cell/collagen interface. At later times, cells exert mechanical pulling forces, resulting in the formation of radial filament arrays (Kopanska et al., 2016; Staneva et al., 2018). **(B).** In the absence of external mechanical forces, collagen networks self-polymerize into overlapping isotropic networks (left). Cell clusters seeded on top of collagen networks exert inward-facing in-plane stresses near the cluster edge and downward-facing stresses near the cluster center, which are balanced by upward-facing stresses around the cluster periphery (right). The mechanical forces exerted by the cell cluster results in local stress and strain gradients on the collagen network that decrease further away from the cluster. The details of the how stress, strain and fiber alignment decay as a function of distance from the cluster are currently not understood and present an interesting topic for future studies.

Cells and groups of cells can exert both puling and pushing forces on ECM networks (Kopanska et al., 2016; Linde-Medina and Marcucio, 2018). For cell spheroids embedded in 3D collagen networks, spheroids initially push on the collagen network, leading to filament compaction and an increase in density at the cell/collagen interface. Later, active pulling forces from cells lead to the generation of radial fiber arrays that are important for invasion of single cells into the collagen network (Figure 1A) (Kopanska et al., 2016; Staneva et al., 2018). For cell clusters migrating collectively on top of collagen networks, inward-facing radial traction forces near the cluster edge induce an in-plane extension of the collagen network in regions surrounding the cells and in-plane network compression in the region directly under the cell or cell cluster. At the same time, downward-facing tractions in the middle of the cluster lead to out-of-plane compression near the cluster center, which is balanced by lower magnitude upward facing forces near the cluster periphery (Figure 1B) (Clark et al., 2020; Clark et al., 2022). The downward-facing pushing force, which acts perpendicular to the primary orientation of filaments, together with a local increase in the collagen density, reduces filament mobility and can therefore lead to an increase in matrix stiffness, though these effects will depend on the relative timescales of the cell-induced strains and collagen network rearrangement. In some situations, like invadopodia formation, these forces are associated with local proteolytic degradation of the collagen network by matrix metaloproteases (MMPs) (Ferrari et al., 2019). MMP secretion can also lead to local degradation of collagen networks and can modulate single cell or collective migration (Wolf et al., 2013; Haeger et al., 2014).

Several studies using different force measurement methods indicate that cells generate mechanical forces on 3D ECM networks on the order of ∼10–100 nN (Hall et al., 2016; Steinwachs et al., 2016; Bashar et al., 2021). Secondary inter-filament bonds in collagen networks, such as electrostatic and hydrophobic bonds, can be broken by forces of ∼20 pN, while forces of 3 nN can induce stretching of single collagen filament up to strains of 20% (Gautieri et al., 2012). This suggests that cell-generated mechanical stresses are indeed sufficient to reorganize collagen networks. However, more precise multiscale modeling is required to make more definite predictions, as length-scales for cellular force measurements and collagen filament and network behavior may not always be comparable. During migration, cells exert mechanical stress and induce matrix strain in a cyclical manner as individual adhesions assemble, transmit cellular stresses on the network and then turn over. Such periodic stress-relaxation cycles, which occur on timescales of minutes to tens of minutes, can lead to a gradual reduction in filament mobility and residual accumulation of stress in the network, and this process occurs at timescales of hours (Pryse et al., 2003; Nam et al., 2016; Pajic-Lijakovic and Milivojevic, 2020). In order to understand the scenario of the filament mobility reduction, it is necessary to consider multi-scale nature of the viscoelasticity of collagen I network.

### Mechanisms Regulating Collagen-I Network Viscoelasticity

**FIGURE 2:**
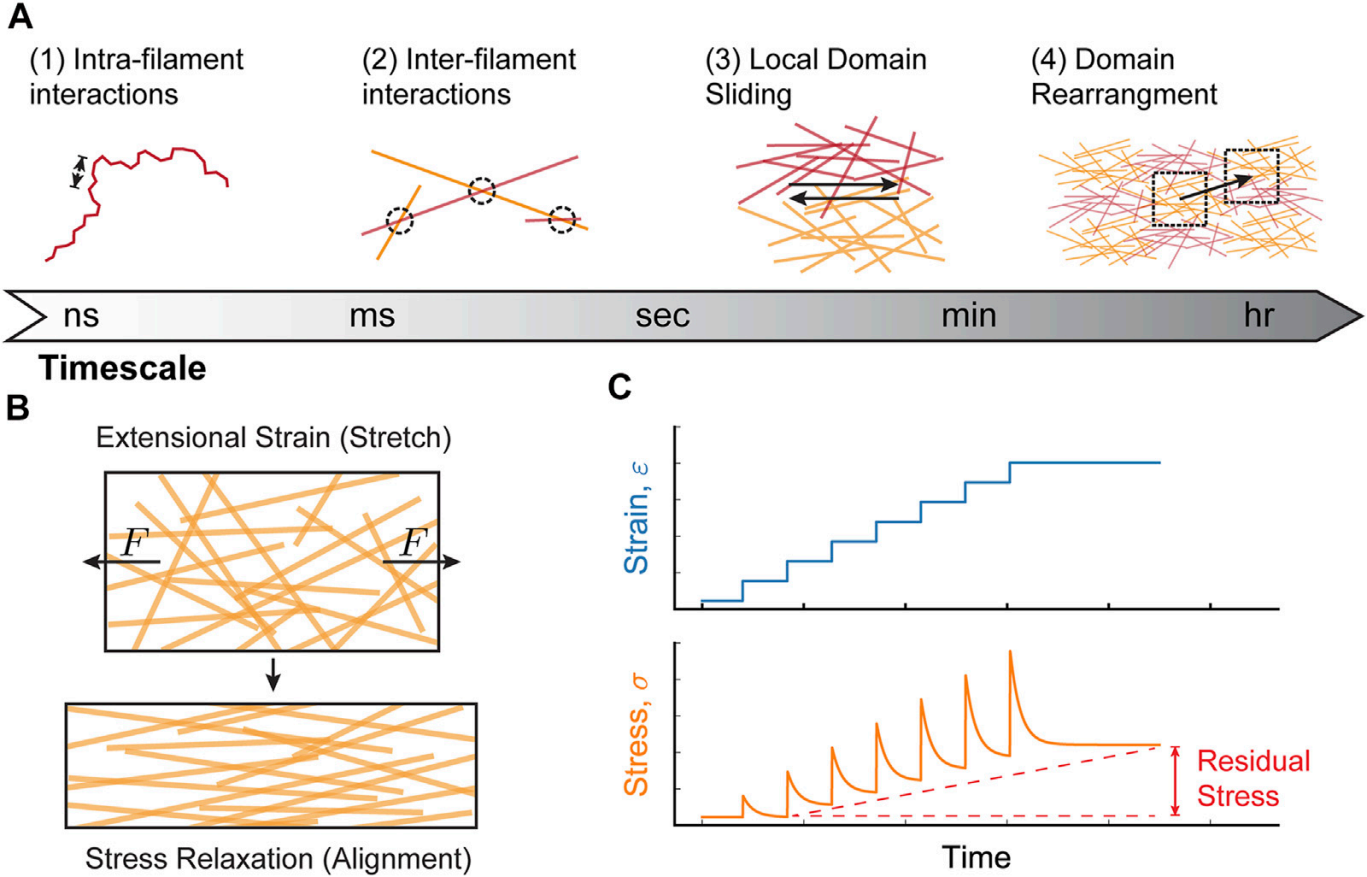
Molecular mechanisms of collagen-I network viscoelasticity. **(A)** Viscoelastic behavior in collagen-I networks arises from interactions at different timescales: (1) nano-to millisecond timescales are dominated by interactions between subunits on an individual filament (intra-filament interactions over short length scales [arrows]; e.g., bending, stretching, twisting), (2) inter-filament interactions (between different filaments) occurs at millisecond-second timescales and includes bond breakage/formation and alignment, (3) at minute timescales, local domains of filaments slide relative to one another, and (4) on tens of minutes to hours, larger domains rearrange within the network. **(B)** When a constant extensional strain (stretch) is applied to a collagen network, the stresses quickly relax due to filament alignment. **(C)** Repeated cycles of extensional strain can result in a gradual accumulation of residual stress. Viscoelastic network behavior can contribute to this phenomenon by preventing complete relaxation after each strain cycle. This behavior is expected during collective cell migration, where strain changes occur on time-scales of hours, while stress relaxation occurs on time-scales of minutes.

Collagen-I networks behave in a viscoelastic manner. This means that when external strain is kept constant, the network will rearrange, relaxing the network stress over time (Pryse et al., 2003; Nam et al., 2016; Elosegui-Artola, 2021). In general, viscoelastic behavior arises from interactions within materials that occur on different timescales. The viscoelastic properties of collagen-I networks can be considered within four main time-scale regimes based on the expected structural changes to the network: 1) nanoseconds to millseconds: intra-filament interactions (i.e. interactions between collagen monomers within a single collagen fiber) caused by single fiber conformations 2) seconds: inter-fiber interactions (i.e. interactions between fibers) and alignment of fibers within mesoscopic domains 3) minutes: sliding of network domains with respect to one another, and 4) tens of minutes to hours: rearrangement of network domains (Figure 2A) (Pryse et al., 2003; Legant et al., 2009; Gautieri et al., 2012; Nam et al., 2016).

Viscoelastic behavior is often measured using two types of rheological experiments: 1) a creep test, where a material is placed under constant stress and the resulting deformation (strain) is measured over time or 2) a stress relaxation test, where a constant strain is applied and stress is measured over time (Nam et al., 2016; Wu et al., 2018; Clark, 2021). In stress relaxation tests, collagen-I networks relax relatively quickly due to structural reorganization of the network (Nam et al., 2016). For example, network stretching results in an alignment of collagen fibers along the stretch axis that relaxes the stresses over time (Figure 2B). However, repeated extensional strain cycles can induce accumulation of residual stress within the network. Network viscoelasticity can contribute to this phenomenon, as the network does not have sufficient time to relax before the next round of applied strain, leading to a gradual increase in network stiffness (Figure 2C). In successive stress relaxation cycles of collagen networks under uni-axial extensional strain, while the residual stress was only ∼5 Pa after the first relaxation cycle, the residual stress increased up to ∼35 Pa after the third relaxation cycle, clearly demonstrating that stresses accumulate in response to periodic stress (Pryse et al., 2003). In order to determine precisely how stress accumulation depends on viscoelastic relaxation, and not purely elastic strain stiffening, more experiments exploring the frequency dependence of cyclic strain will be required.

Collagen-I network strain stiffening and the associated structural changes to the network is a multi-scale phenomenon. Strain increases at short time-scales (regimes 1 and 2) induce entropic (conformational changes and filament straightening) and enthalpic (mostly filament stretching) effects in individual filaments, leading to overall network stiffening. However, strain stiffening is relatively short-lived in collagen gels, and even for high strain rates, the stiffness returns to basal levels within ∼5 min (Nam et al., 2016). This relaxation is likely due to breaking of inter-filament bonds and filament alignment along the stress axis (Nam et al., 2016). However, periodic strain of collagen networks in response to cell-mediated stresses, which occur on time-scales of minutes to hours (regimes 3 and 4) can lead to long time-scale and more permanent stiffening caused by the accumulation of residual stress during periodic contractions (Pryse et al., 2003; Xu et al., 2013; Pajic-Lijakovic and Milivojevic, 2020).

Collagen-I networks have important similarities and differences with other well-studied biological networks, such as cytoskeletal networks of actin filaments. Many of the qualitative features of actin network behavior, including the different timescales between intra- and inter-filament interactions, entropic vs enthalpic considerations of network deformation and strain stiffening behavior (Gardel et al., 2004b; 2004a), also apply to collagen-I networks. However, collagen fibers are much more flexible than actin filaments. Actin filaments have a persistence length (*L*_*p*_) of ∼18 μm, similar to their typical contour length in cells (*L*_*p*_ *≈ L*_*c*_), which classifies actin filaments as semi-flexible polymers (Sanghvi-Shah and Weber, 2017). Collagen fibers, on the other hand have a persistence length of ∼14–180 nm, orders of magnitude shorter than their contour length in a physiological setting (*L*_*p*_ *<< L*_*c*_), classifying them as flexible polymers (Ghavanloo, 2017). Consequently, the cumulative effects of fiber bending and stretching plays a larger role for actin networks compared to collagen networks, where inter-fiber interactions are dominant. Such inter-fiber interactions are mediated by fiber length and crosslinking density as well as network anisotropy and the direction of external strain with respect to fiber orientation (Fraley et al., 2015; Nam et al., 2016; Ghavanloo, 2017; Sarker et al., 2019). It should also be noted that fiber bundling within networks, which may differ between actin and collagen networks, can drastically change persistence length, as the flexural rigidity of fibers is highly dependent on fiber radius (Howard, 2001). Moreover, while actin networks can be treated as isotropic and incompressible under long time-scale deformation, with a Poisson’s ratio of ∼0.5 (Mokbel et al., 2020), collagen-I networks are anisotropic and induce a volume increase under multi-axial deformation, with a Poisson’s ratio of ∼1.7 (Ban et al., 2019). This anomalous behavior of collagen networks is a consequence of the strain stiffening and fiber alignment in response to strain.

### Experimental Measurements of Collagen Network Viscoelasticity

Several studies probing the viscoelastic properties of collagen gels have shown differences in residual stress accumulation depending on the nature of the applied strain (i.e., shear, stretch or compression). The extensional modulus of collagen-I networks is several times larger than the compressional modulus under the same absolute strain (Achilli and Mantovani, 2010), meaning that networks are easier to compress than to stretch. Additionally, compression induces network disordering accompanied with entropic effects caused by single-chain conformational changes, while extension enhances the chain alignment and single-chain stretching, leading to a higher storage modulus. Shear strains ensure sliding of collagen meso-domains over each other, which minimizes matrix residual stress accumulation (Nam et al., 2016). Extensional strains, on the other hand, induce more intense residual stress accumulation compared with similar magnitude shear strains due to the fact that stretched collagen filaments store more energy than compressed filaments under the same absolute strain (Broedersz and MacKintosh, 2014).

Collagen networks can also be considered as poro-elastic, as fluid can flow within the network mesh in response to strain (Chandran and Barocas, 2004). Network pore size can affect the compressibility of collagen networks by affecting water permeability (Ban et al., 2019). For larger particles, diffusivity is controlled by the relative sizes of the molecules and network pores (Burla et al., 2020b). However, work investigating connective tissues made primarily of collagen-I networks found that collagen crosslinking increased storage and loss moduli without affecting extracellular diffusive water transport, suggesting that fluid flow is not a major determinant of collagen network rheology (Sauer et al., 2019). Additionally, fluid flow through collagen networks woud be expected to occur within seconds, while viscoelastic effects induced by collective cell migration typically take place on significantly longer time-scales.

Experimental evidence suggests that strain stiffening and stress accumulation also depend on the strain magnitude and the number of repeated stress relaxation cycles (Pryse et al., 2003; Xu et al., 2013; Licup et al., 2015; Nam et al., 2016). At low shear stresses (<∼10 Pa), collagen networks are linearly elastic, while at higher stresses (>10 Pa) collagen networks display dramatic non-linear strain stiffening (Licup et al., 2015). At even higher stresses, collagen networks can also fracture, and the strain at which networks fracture depends strongly on their connectivitity, but not single fiber properties (Burla et al., 2020a). In addition, high shear strains result in residual stress accumulation, while there is essentially no stress accumulation following low shear strain (Nam et al., 2016). Extensional stress relaxation tests in collagen gels have shown that higher initial strains lead to increased stress relaxation rates, suggesting that the viscoelastic relaxation is also affected by strain magnitude (Xu et al., 2013). Stress magnitude is an important consideration in single-cell vs collective migration, as larger groups of cells exert higher mechanical force on their substrate, resulting in more extensive network reorganization (Mertz et al., 2012; Clark et al., 2020; Clark et al., 2022).

The organization and topological properties of collagen-I networks can significantly impact bulk rheological properties (Nam et al., 2016). Studies using *in vitro* polymerized collagen gels have demonstrated that varying polymerization conditions, for example by changing collagen monomer concentrations, polymerization temperature or pH, results in substantial changes in network organization. Increasing collagen monomer concentration, polymerization temperature or pH all lead to collagen networks with shorter filaments and smaller mesh sizes (i.e. higher density) (Wolf et al., 2013; Fraley et al., 2015; Sapudom et al., 2015; Burla et al., 2020a). Collagen networks with higher monomer concentrations are not only denser, but also significantly stiffer (Paszek et al., 2005). However, this may only hold for low stresses; due to the strain stiffening behavior of collagen networks, at high stresses (>10 Pa, on the order that can also be induced by cell movements), network stiffness no longer depends on collagen concentration (Licup et al., 2015). This interesting result suggests that induced domain sliding is the dominant mechanism of structural changes in the network under high stress. While increasing polymerization temperature leads to denser collagen networks, these networks are significantly softer (Guzman et al., 2014). It is therefore important to consider the independent effects of collagen network topology and bulk rheological properties, as these properties can also have different effects on cell behavior. Future studies using recently developed synthetic matrices whose topology, viscoelastic properties and biochemical functionalization can be more precisely tuned will be crucial to understanding the effects of these independent parameters (Dietrich et al., 2018; Yang et al., 2021; Elosegui-Artola et al., 2022).

### Effects of Collagen-I Network Properties on Cell Behavior

Just as cellular mechanical forces can induce changes in collagen-I network organization and mechanical properties, modifications in network topology and mechanics can influence cellular behavior. Collagen-I networks are complex materials, and it is difficult to independently tune elastic and viscoelastic properties. Previous studies using hydrogels such as poly-a-acrylamide have been widely used to test the effects of substrate elasticity. Cells and groups of cells typically develop more mature adhesions and exert higher forces on stiffer elastic substrates, leading to faster spreading and a biphasic change in migration speed (Peyton and Putnam, 2005; Bangasser et al., 2017). More recent studies have shown that tuning substrate viscoelasticity can also influence spreading and migration dynamics (Murrell et al., 2011; Cantini et al., 2012; Charrier et al., 2018; Chaudhuri et al., 2020; Mandal et al., 2020). Small clusters of cells have recently been shown to exhibit spontaneous persistent migration on viscoelastic collagen-I networks, and reducing the viscoelastic relaxation time leads to reduced migration persistence. Single cells, on the other hand, are not able to migrate persistently due to their limited ability to deform collagen networks, again highlighting the importance of stress magnitude in collagen network rheology (Clark et al., 2020; Clark et al., 2022).

In addition to the rheological properties of collagen-I networks caused by cell movement, local network topology can also regulate cell behavior. In particular, collagen fiber alignment has been shown to determine migration directionality. While cells embedded in isotropic collagen-I networks display overall random migration, when the networks are stretched to align filaments, cells preferentially migrate along the axis of collagen fiber alignment (Riching et al., 2014). Even in isotropic networks, at cellular length-scales, migration direction is influenced primarily by local collagen fiber orientation and collagen fiber diameter, but not by bulk rheological properties (Fraley et al., 2015; Sapudom et al., 2015). Collagen network topology has also been shown to modulate transitions between single-cell and collective migration. Longer and more mobile collagen fibers allow cells to easily modulate local substrate stiffness gradients and on that base favor collective migration (Sarker et al., 2019). In larger groups of cells embedded in 3D collagen networks, increased network density (i.e. smaller pore sizes) can favor multicellular migration modes, and cell-mediated fiber alignment can also enhance cell invasion into the network (Haeger et al., 2014; Kopanska et al., 2016; Ilina et al., 2020; Kang et al., 2021).

Asymmetries in network deformation can lead to the formation of gradients in ECM networks. It is well described that elastic stiffness gradients lead to a single- and collective migration in the direction of higher stiffness, termed “durotaxis” (Lo et al., 2000; Raimon et al., 2016). Evidence for “negative” or “inverse durotaxis” (migration away from regions of higher stiffness) has also emerged (Ueki and Kidoaki, 2015; Thompson et al., 2019; Isomursu et al., 2020; Oliveri et al., 2021). Currently, it is still unclear how cells respond to such rheological gradients in viscoelastic ECM networks, although recent work suggests that cell clusters may exhibit negative durotaxis during collective migration on collagen networks (Clark et al., 2020; Clark et al., 2022).

ECM network viscoelasticity can also impact invasive behavior from larger groups of cells, which is particularly important in the context of cancer dissemination. For cancer cell spheroids embedded in 3D collagen matrices, crosslinking collagen fibers, which leads to higher stiffness and reduced viscoelastic relaxation, leads to a reduction in cell invasion into the surrounding matrix. However, addition of the crosslinker after invasion has already begun further increases invasion, underlining the importance of the timing of network modification in affecting cellular behavior (Staneva et al., 2018). Similarly, varying the viscoelastic properties of functionalized synthetic matrices can control tissue branching and invasion of breast epithelial cells (Elosegui-Artola et al., 2022). Recent work has also found that pressure-driven flows, caused by cell swelling at the spheroid-gel biointerface, can also drive collective migration from spheroids in collagen gels, suggesting that there may still be additional mechanisms contributing to local invasion (Raghuraman et al., 2022).

## Conclusions and Outlook

Viscoelastic behavior of the collagen-I networks during collective cell migration is determined by multi-scale molecular events occurring over serval different timescales. Cellular mechanical forces are sufficient to induce structural changes in collagen networks and can lead to rheological changes such as strain stiffening and residual stress accumulation, which depend on the magnitude and type of strain as well as the rate of strain change. In collagen networks, these effects are controlled primarily by changes in filament mobility within the network.

In addition to cell-mediated changes to collagen networks, the rheological properties of collagen networks can also influence cell behavior, in particular during single-cell and collective migration. The experimental modification of network rheology offers new opportunities to study the interplay between cell-matrix interactions. Future studies probing the role of collagen network modification and the corresponding changes in cell behavior will have important implications for therapies targeting pathologies related to changes in cell and tissue dynamics, such as cancer metastasis.
